# Spermine Suppresses Adipocyte Differentiation and Exerts Anti-Obesity Effects In Vitro and In Vivo

**DOI:** 10.3390/ijms231911818

**Published:** 2022-10-05

**Authors:** Sachie Nakatani, Yasuhiro Horimoto, Natsumi Nakabayashi, Mayumi Karasawa, Masahiro Wada, Kenji Kobata

**Affiliations:** Graduate School of Pharmaceutical Sciences, Josai University, Sakado, Saitama 350-0248, Japan

**Keywords:** polyamine, spermine, differentiation, adipocyte, obesity

## Abstract

Endogenous polyamines such as putrescine (Put), spermidine (Spd), and spermine (Spm) affect adipocyte differentiation. In this study, we investigated the effect of exogenously supplemented polyamines on mouse adipocyte differentiation and anti-obesity actions in vitro and in vivo. The preadipocyte cell line, 3T3-L1, was cultured with Put, Spd, or Spm, and lipid accumulation in the cells was measured by Oil Red O staining. Lipid accumulation was significantly suppressed by Spm. Suppression of *CCAAT/enhancer binding protein α* mRNA by Spm suggested that the decreased lipid accumulation was due to delaying the cell differentiation. The body weight and fat of obese mice induced with a high-fat diet were reduced by oral ingestion of Spm. In conclusion, oral supplementation of Spm has the ability to prevent obesity through inhibition of adipocyte differentiation.

## 1. Introduction

Polyamines such as putrescine (Put), spermidine (Spd), and spermine (Spm) exist in all living cells. Due to their cationic nature, they can interact with negatively charged macro-molecules such as DNA, RNA, proteins, and phospholipids [1]. These polyamines have multiple functions in cell proliferation, differentiation, and DNA methylation [1,2,3,4]. The role of polyamines and the mechanisms by which they are involved in cell differentiation depend on the cell type. Intracellular polyamines are essential for the differentiation of preadipocytes into adipocytes, and the depletion of polyamines by inhibiting their synthesis suppresses the differentiation of these cells [5,6,7]. Spd, but not Spm, is especially essential for adipogenesis [7]. Natural polyamines are readily interconvertible inside the cells. Our previous study showed that changes in the intracellular Spm and Spd levels by controlling their syntheses also affect adipocyte differentiation [8]. Conversely, another report showed exogenous polyamines decelerate adipogenic differentiation from human bone marrow-derived mesenchymal stem cells [9]. Spd/Spm N-1-acetyltransferase transgenic mice are characterized by a compensatory increase in polyamine biosynthetic enzyme activity. These mice exhibit attenuated insulin production, decreased white adipose tissue, and a distinctly lean phenotype [10,11]. Mice treated intraperitoneally with Spm exhibit improved glucose utilization associated with enhanced fat oxidation and a loss of white adipose mass [12]. Therefore, the specific roles of individual polyamines during adipogenesis have remained ambiguous.

Intracellular polyamine contents in some tissues decreased with increasing age [13,14]. Polyamines are supplied to the body in three ways: biosynthesis in human cells, production in the intestinal flora, and oral uptake from food [15]. Polyamines are found in all types of food and in a wide range of concentrations [14,16]. There are some reports on dietary supplementation for the age-related decrease in polyamine synthesis in vivo. For example, a polyamine-rich diet decreases age-associated mortality in mice [17]. Another report showed intake of polyamine activates cells in the whole body and provides an anti-aging effect [18].

Although food is one of the main sources of polyamines for organisms, the effect of orally administered polyamines on adipocyte differentiation is not clear. In this study, we investigated the effect of exogenously supplemented polyamines on adipocyte differentiation in vitro and their influence on mouse adipose tissue in vivo.

## 2. Results

### 2.1. Spermine Suppressed Lipid Accumulation in 3T3-L1 Cells

**Figure 1:**
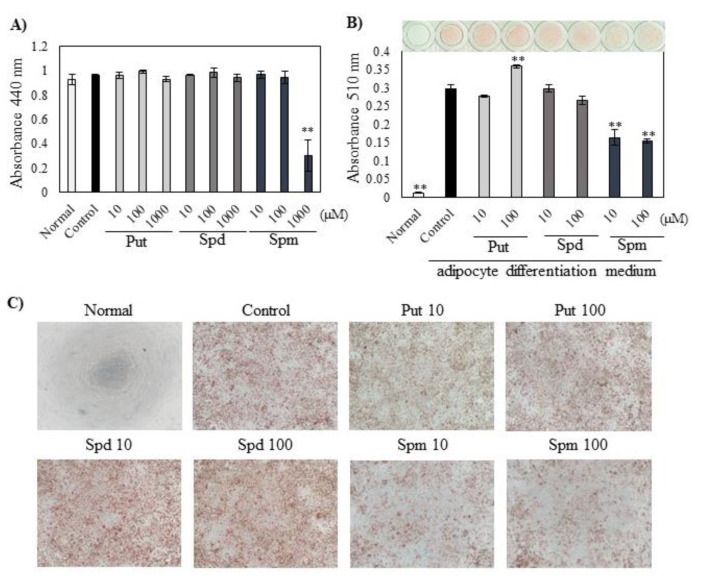
Effect of polyamines on (**A**) cell viability (**B**,**C**) lipid accumulation in 3T3-L1 adipocytes. (**A**) In the cell viability experiment, 1 mM aminoguanidine alone was used as a control without adding polyamine. (**B**) Red-stained lipid droplets by oil red O staining were dissolved, and the absorbance was measured. (**C**) Images of treated cells obtained using an inverted microscope (×40). Red spots in images represent areas stained by Oil Red O. Data are presented as means ± S.E. derived from three independent experiments; each experiment carried out in 3 or 4 culture wells. Dunnett’s test, ** *p* < 0.01 vs. control.

Put and Spd did not affect the viability of 3T3-L1 cells up to the highest concentration we tested (1000 µM). Spm had no toxicity up to 100 µM (Figure 1A). In the suppressive ability of each polyamine on lipid accumulation at nontoxic concentrations (10 and 100 µM), Spm significantly suppressed lipid accumulation to about half of the control even at a lower concentration (10 µM) (Figure 1B,C).

### 2.2. Spermine Inhibited Differentiation of Preadipocytes to Adipocytes

**Figure 2:**
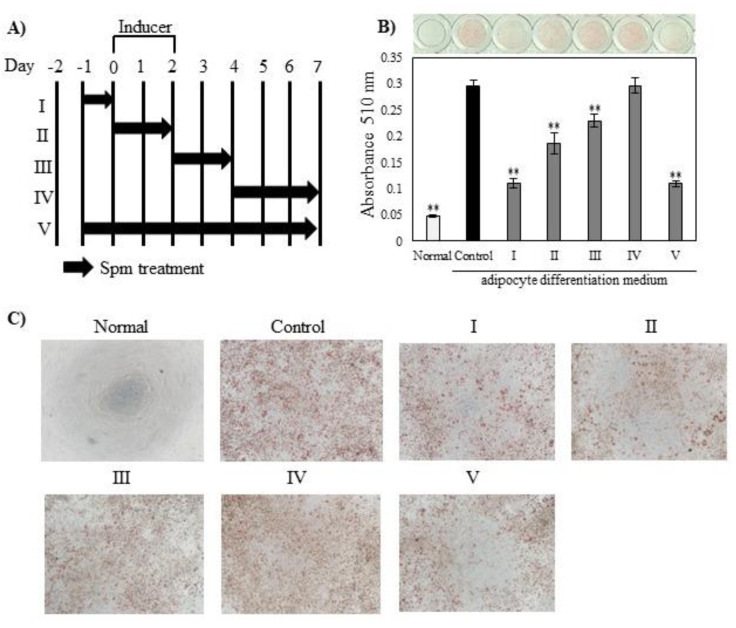
Spm inhibits differentiation of 3T3-L1 cells: (**A**) Description of Spm addition schedule; (**B**) Effect of Spm on lipid accumulation in 3T3-L1 adipocytes. Representative photographs showing cell monolayers stained with Oil Red O. (**C**) Images of treated cells obtained using an inverted microscope (×40). Red spots in images represent areas stained by Oil Red O. Data are presented as means ± S.E. derived from three independent experiments; each experiment carried out in 4 culture wells. Dunnett’s test, ** *p* < 0.01 vs. control.

In order to determine when 100 µM Spm addition affected lipid accumulation, we adjusted the timing of its addition to 3T3-L1 cells (Figure 2A). When Spm was added only from day −1 to day 0, lipid accumulation was remarkably suppressed to about 33% of control (Figure 2B), showing the same inhibitory effect as the addition of Spm in all the time periods (V). Simultaneous addition of Spm with the adipocyte differentiation medium (inducer) (II), and addition after induction (III), attenuated the inhibitory effect. However, even later additions of Spm (IV) had no effect.

### 2.3. Spermine Suppressed the Expression of Adipocyte Differentiation-Related Gene mRNAs

**Figure 3:**
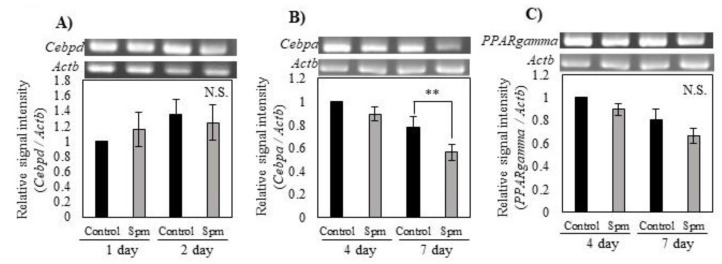
Effect of Spm on the expression of differentiation-related genes in 3T3-L1 cells: (**A**) Expression of *Cebpd* mRNA on days 1 and 2 of culturing in differentiation medium; (**B**) Expression of *Cebpa* mRNA on days 4 and 7 of culturing in differentiation medium; (**C**) Expression of *PPARgamma* mRNA on days 4 and 7 days of culturing in differentiation medium. Data are shown as means ± S.E. derived from three independent experiments; each experiment was carried out in 3 culture wells. Statistics were compared between C group and Spm group by day. Student’s *t*-test, ** *p* < 0.01.

We, therefore, investigated the effect of Spm on expression levels of adipogenesis-related transcription factor mRNAs by reverse transcription polymerase chain reaction (RT-PCR). A significant decrease in the *CCAAT/enhancer binding protein (C/EBP)*, *alpha* (*Cebpa*) mRNA level resulted from the addition of Spm on day 7 (Figure 3B). *Peroxisome proliferator-activated receptor gamma* (*PPARgamma*) mRNA also showed a tendency to be suppressed to about 75% of control by the addition of Spm (Figure 3C). In contrast, Spm did not affect the mRNA level of *CCAAT/enhancer binding protein delta* (*Cebpd*) (Figure 3A).

### 2.4. Orally Intake of Spermine Showed Anti-Obese Effects in Mice

**Figure 4:**
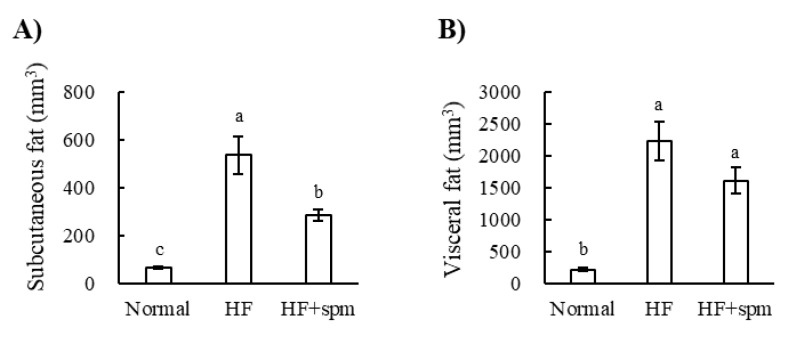
Effect of Spm on the ingestion fat volume scanned by micro-CT system: (**A**) Subcutaneous fat; (**B**) Visceral fat. Data are shown as means ± S.E. Tukey’s test, *p* < 0.05. Values in the same figure not sharing a common letter avove the bar are significantly different one another.

Spm was added to the high-fat diet at 0.1% (HF + Spm) and fed to mice for 12 weeks. The results are summarized in Table 1 and Figure 4. There was no significant difference in caloric intake between HF and HF + Spm. The body weight and blood triglyceride levels of mice fed HF + Spm were significantly lower than those of mice fed HF (Table 1). The volume of the subcutaneous fat was also lower in HF + Spm than in HF (Figure 4A). The other indices related to obesity, such as white adipose tissue (WAT) weight, blood cholesterol and description of ingestion status, organ weights, and serum parameters fat volume, tended to be suppressed by the Spm supplementation.

## 3. Discussion

This study reported a relationship between Spm and adipocyte differentiation in vitro and in vivo. First, we evaluated the effects of polyamines, Put, Spd, and Spm on toxicity and adipogenesis in 3T3-L1 cells. The 3T3-L1 preadipocytes have the ability to differentiate into mature adipocytes and can reproduce the differentiation process from preadipocytes to mature adipocytes. In the suppressive ability of each polyamine on lipid accumulation at nontoxic concentrations, Spm significantly suppressed lipid accumulation (Figure 1B). Lee et al. reported that supplementation of the culture medium with Put, Spd, and Spm suppressed adipogenic differentiation and lipid accumulation of hBMSCs [9]. Ishii et al. reported that increasing intracellular Spm reduced lipid accumulation of 3T3-L1 cells [8]. In the present study, supplementation with Spm, not Put or Spd, suppressed lipid accumulation. Although Spd was reported to be essential for the formation of mature adipocytes, the effect of Spm on adipocyte differentiation was ambiguous. These results suggested that excessive amounts of Spm might be taken into cells and function as a suppressor of lipid accumulation.

In order to determine when Spm affected lipid accumulation, we adjusted the timing of its addition to 3T3-L1 cells (Figure 2A). The addition of Spm for just one day at the preadipocyte stage significantly suppressed lipid accumulation (Figure 2B), showing the same inhibitory effect as the addition of Spm in all the time periods. 

An excessive supply of polyamines enhances the metabolic system (i.e., Spd/Spm N1-acetyltransferase (SSAT)) in cells, and then the enhancement of SSAT activity depletes acetyl-CoA, thereby facilitating β-oxidation [11,19,20]. The suppression of lipid accumulation by Spm observed in our in vitro study has two potential causes: facilitation of β-oxidation and suppression of differentiation of preadipocytes. 

The differentiation of preadipocytes into adipocytes is regulated by a network of transcription factors that control expressions of many hundreds of proteins [21]. PPARgamma and C/EBP are known as the master regulators of adipogenesis. Early on in adipocyte differentiation, the Cebpd is rapidly induced to express and later activate PPARgamma [22]. Cebpa is induced after the induction of PPARgamma but preceding the synthesis of many of the protein characteristics of differentiated cells during adipogenesis [23]. Our results showed that Spm suppressed the early stage of adipocyte differentiation, namely the differentiation of preadipocytes to mature adipocytes. The 3T3-L1 cells have the ability to accumulate lipids through the expression of transcription factors related to adipose differentiation [24]. We, therefore, investigated the effect of Spm on expression levels of adipogenesis-related transcription factor mRNAs by RT-PCR. A significant decrease in the *Cebpa* mRNA level and a tendency to decrease *PPARgamma* mRNA levels resulted from the addition of Spm (Figure 3). In contrast, Spm did not affect the mRNA level of *Cebpd* (Figure 3A). Increases in PPARgamma and Cebpa are important events in the course of 3T3-L1 cell differentiation [23]. Therefore, Spm may delay the differentiation of preadipocytes by suppressing genes involved in this process. Our results are consistent with data showing that Spm reduced the expression of *PPARgamma* mRNA in hBMSCs [9].

Because the extracellularly supplemented Spm suppressed adipogenesis in vitro, we examined the influence of orally ingested Spm in mice fed a high-fat diet. The present study showed that concomitant oral administration of Spm to mice fed a high-fat diet suppressed the increases in body weight, blood triglycerides, and visceral fat induced by the high-fat diet (Table 1, Figure 4A). Sadasivan et al. reported that an intraperitoneal dose of 10 mg/kg per day of Spm to mice fed a high-fat diet resulted in a 24% reduction in body weight and a 57% loss of white adipose mass compared to untreated controls [12]. Our study showed that dietary Spm supplementation, which is non-invasive, contributes to the reduction in body weight, visceral fat, and blood triglyceride. 

Recently, some health benefits of oral ingestion of polyamines have been reported. Soda et al. reported the effects of a polyamines-rich diet on extending healthy lifespans in mice [17]. In recent years, research on Spd among polyamines as supplements has accelerated. Madeo et al. reported that Spd acts as a caloric restriction mimetic and extends a healthy lifespan through autophagy [25]. A randomized, double-blind, placebo-controlled trial reported improvement in memory performance after 1200 mg of spermidine per day for 3 months [26]. While there are many reports on Spd, there are few reports on oral intake of Spm. Our data provided a basic study on the benefits of Spm supplementation.

## 4. Materials and Methods

### 4.1. Cell Culture

The 3T3-L1 preadipocytes (RIKEN Bio Resource Research Center, Tsukuba, Japan) were cultured and maintained in Dulbecco’s modified Eagle’s medium (DMEM; Thermo Fisher Scientific K.K., Tokyo, Japan) containing 3.7 g/L sodium bicarbonate (Fujifilm Wako Pure Chemical, Osaka, Japan), 50 IU/mL penicillin–50 µg/mL streptomycin (Meiji Seika Pharma, Tokyo, Japan), and 10% fetal bovine serum (FBS; Fujifilm Wako) as a normal medium.

### 4.2. Cell Viability

The 3T3-L1 cells were plated in 96-well plates at a density of 1.5 × 10^4^ cells/well and incubated at 37 °C. One day after plating, the cells were treated with a culture medium containing 10, 100, and 1000 µM polyamines (Sigma-Aldrich Japan, Tokyo, Japan). Polyamines were added with 1 mM aminoguanidine (Sigma-Aldrich Japan). After 24 h, 10 µL/well WST-1 regents (Roche Diagnostics K.K., Tokyo, Japan) were added to the cells prior to incubation for 1 h. Formazan dye transformation was quantitated by measuring the absorbance at 440 nm using a Spectra Max M2e microplate reader (Molecular Devices, Tokyo, Japan). 

### 4.3. Adipocyte Differentiation

The 3T3-L1 cells were seeded at 1.5 × 10^4^ cells/well in 96-well plates with a growth medium. One day after seeding, polyamines were added with 1 mM aminoguanidine. Cells to which only aminoguanidine was added were used as a control. Two days after seeding, the cells were incubated with an adipocyte differentiation medium (5% FBS and 0.035 g/L D-(+)-glucose in DMEM with 0.5 mM 3-isobutyl-1-methylxanthine, 1 µM dexamethasone, and 1 µg/mL insulin (all from Sigma-Aldrich Japan) for 2 days. After a subsequent 5 days of incubation in a second adipocyte differentiation medium (5% FBS and 0.035 g/L D-(+)-glucose in DMEM with 1 µg/mL insulin), the cells were semi-fixed with 10% formaldehyde for 20 min, followed by complete fixation with 20% formaldehyde for 20 min. After washing with distilled water for 5 min, cellular lipids were stained with a 0.3% Oil Red O solution in isopropyl alcohol:water (60:40 (*v*/*v*)) for 1 h at room temperature. After again washing with distilled water, differentiation was monitored by extracting the stained lipids with isopropanol. The absorbance of the isopropanol extract was measured at 510 nm with a Spectra Max M2e microplate reader. The timing of polyamines treatment is shown in Figure 2A. 

### 4.4. RNA Extraction and the Semiquantitative Reverse Transcription Polymerase Chain Reaction (RT-PCR)

Total RNA was extracted from 3T3-L1 cells with RNAiso Plus (Takara Bio, Kusatsu, Shiga, Japan). RNA samples with OD260/280 ratios of about 2.0 were used for semiquantitative RT-PCR. cDNA was prepared from 1 µg of total RNA by using the Superscript First-Strand Synthesis System (Thermo Fisher Scientific, K.K.). Amplification was performed in a 10 µL reaction mixture containing 1 µL of the cDNA solution using EX Taq (Takara Bio). Primers for Actb (NM_007393), PPARgamma (NM_011146), Cebpd (BC139293), and Cebpa (NM_007678) were as follows: Actb, 5′-GGCTCCTAGCACCATGAAGA-3′, 5′-GTACTCCTGCTTGCTGATCCA-3′; PPARgamma, 5′-CATTCCATTCACAAGAGCTG-3′, 5′-ACCTGATGGCATTGTGAGAC-3′; Cebpd, 5′-CTCCCGCACACAACATACTG-3′, 5′-AGAGGCAACGAGGAATCAAG-3′, and Cebpa, 5′-GCCGAGATAAAGCCAAACAA-3′, 5′-GACCCGAAACCATCCTCTG-3′. PCR products were electrophoresed on 2% (*v*/*v*) agarose gels (Takara Bio), stained with ethidium bromide (Sigma Aldrich), and photographed. All gels were digitally imaged and analyzed using a Gel Doc™ EZ Imager (BIO RAD, Hercules, CA, USA). Band intensities of these digitally imaged gels obtained were determined using ImageJ ver. 1.51 software (National Institutes of Health, MD, USA) from at least three different experiments. The signals were normalized by those of Actb transcripts.

### 4.5. Animals

Thirty, five-week-old male C57BL/6J mice were purchased from CLEA Japan, Inc. (Tokyo, Japan). The mice were housed in individual cages in a temperature- and humidity-controlled room with a 12 h light/dark cycle. After a one-week acclimatization, the mice were divided into three groups: normal diet (n = 10), high fat diet (n = 10), and 0.1% Spm-containing high fat diet (n = 10). For the normal diet, D12450J (Research Diets, Inc., New Brunswick, NJ, USA), a rodent diet with 10 kcal% fat, was used. For the high-fat diet, D12492, a rodent diet with 60 kcal% fat, was used. Spm was mixed with a high-fat diet. All mice were fed for 12 weeks. Then, the mice were anesthetized, and the blood was collected from the inferior vena cava. Liver and testicular peripheral WAT were taken and weighed. Blood was kept for 1 h at room temperature, then centrifuged at 2000× *g* for 20 min. Serum levels of triglycerides, glucose, and total cholesterol were measured by the Triglyceride E-Test, Glucose E-Test, and Cholesterol C II-Test (Fujifilm Wako), respectively. 

### 4.6. Fat Volume

Mice were anesthetized with isoflurane (Pfizer Japan Inc., Tokyo, Japan) before scanning of computed tomography (CT) images by microcomputed tomography (micro-CT) system (CosmoScan GX; Rigaku, Tokyo, Japan). The CT images of visceral and subcutaneous fat were visualized using Analyze 12.0 software (AnalyzeDirect, Inc., Stilwell, KS, USA). The region between the vertebrae L1–L4 was evaluated for fat mass.

### 4.7. Statistical Analyses

Statistical analyses were performed with JMP^®^ software (SAS Institute, Cary, NC, USA). Two groups were compared by Student’s *t*-test. Multiple groups were compared by a one-way analysis of variance, followed by Dunnett’s test and Tukey’s test. Differences were considered as significant at *p* < 0.05 or *p* < 0.01. Data are expressed as means ± standard error.

## 5. Conclusions

In conclusion, supplementation of Spm, both in vitro and in vivo, suppressed the differentiation of preadipocytes, resulting in fat loss in mice. This finding suggests a potential new treatment modality for obesity.

## Figures and Tables

**Table 1:** Description of food intake, organ weights, and serum parameters.

	Normal	HF	HF + Spm
Food consumption (g/day)	2.551 ± 0.029	2.210 ± 0.023	2.156 ± 0.022
Caloric intake (Kcal/day)	9.809 ± 0.112	11.581 ± 0.118	11.298 ± 0.113
Body weight (g)	26.989 ± 0.281 ^c^	37.439 ± 1.698 ^a^	32.955 ± 0.642 ^b^
WAT ^1^ weight (g)	0.556 ± 0.047 ^b^	2.180 ± 0.242 ^a^	1.715 ± 0.203 ^a^
Liver wight (g)	0.993 ± 0.014 ^b^	1.168 ± 0.058 ^a^	1.084 ± 0.029 ^a^
Blood triglyceride (mg/dl)	63.167 ± 4.818 ^ab^	76.833 ± 4.545 ^a^	58.833 ± 4.020 ^b^
Blood glucose (mg/dl)	434.222 ± 18.126 ^b^	517.889 ± 20.434 ^a^	490.933 ± 18.214 ^ab^
Blood cholesterol (mg/dl)	91.25 ± 3.232 ^b^	152.25 ± 12.174 ^a^	129.75 ± 4.684 ^a^

Data are shown as the means ± S.E. Tukey’s test *p* < 0.05. Values with different superscript letters are significantly different. ^1^ WAT: white adipose tissue.

## Data Availability

Not applicable.
